# Performance Evaluation of the New Compound-Carrier-Modulated Signal for Future Navigation Signals

**DOI:** 10.3390/s16020142

**Published:** 2016-01-28

**Authors:** Ruidan Luo, Ying Xu, Hong Yuan

**Affiliations:** 1 College of Materials Science and Opto-Electronic Technology, University of Chinese Academy of Sciences, Beijing 100049, China; luoruidan@aoe.ac.cn; 2 Academy of Opto-Electronics, Chinese Academy of Sciences, Beijing 100094, China; yuanh@aoe.ac.cn

**Keywords:** GNSS, NSCC, tracking performance, multipath effect, modulation scheme

## Abstract

Navigation Signal based on Compound Carrier (NSCC), is proposed as the potential future global navigation satellite system (GNSS) signal modulation scheme. NSCC, a kind of multi-carrier (MC) signal, is generated by superposition and multi-parameter adjustment of sub-carriers. Therefore, a judious choice of parameter configation is needed. The main objective of this paper is to investigate the performance of the NSCC which is influenced by these parameters and to demonstrate its structure characteristics and superiority, employing a comprehensive evaluation system. The results show that the proposed NSCC signal processes full spectral efficiency and limited out of band (OOB) emissions, satisfying the demands of crowed frequency resources. It also presents better performance in terms of spectral separation coefficients (SSCs), tracking accuracy, multipath mitigation capability and anti-jamming reduction compared with the legacy navigation signals. NSCC modulation represents a serious candidate for navigation satellite augmentation systems, especially for signals applied in challenging environments.

## 1. Introduction

Satellite navigation systems and their augmentation systems are fundamental infrastructures for countries, and signal structure design is one of their key links. The navigation signal is precisely a function of the message vehicle and air interface to link up the ground-based infrastructures and users with satellites. Therefore, the signal modulation scheme directly determines the systematic intrinsic performance.

The Global Navigation Satellite System (GNSS) and its augmentation systems are undergoing a process of enhancement and diversification. The ever increasing user requirements in terms of accuracy, robustness, integrity and reliability, are stimulating the evolution towards the future advanced GNSS signals. The overall evolutionary trends focus on spectrum compatibility and resistance to propagation and environmental dagration [1].

The construction of GNSSs is increasingly improving and expanding. Meanwhile, regional augmentation systems and space-based augmentation systems are emerging in succession. It is predicted that by 2030 there will be more than 160 satellites and over 400 signals in space [2]. The already intensive frequency resource will be more crowded and the increasing mutual interference will degrade the systematic accuracy and performance. Better spectrum efficiency will become a critical issue in navigation signal design.

The positioning accuracy is also a primary concern for signal design since the signal strictly determines the systematic natural accuracy limits. The potential performance of the GNSS receiver can only finitely approach the limits, regardless of any development of the technology. Furthermore, due to the navigation channel characteristics, multipath effects are some of the main error sources [3], so the multipath mitigation capability becomes a key design consideration. 

In addition, the fusion of navigation service and communication service has become a development direction of the information industry. Composite designs based on the navigation signals and communication signals have turned into a new solution for future advanced GNSSs.

Binary phase shift keying (BPSK) modulation was the first modulation scheme adopted by satellite navigation because of its simplicity. Due to the coexistence of ever-increasing space signals and its optimal performance, binary offset carrier (BOC) modulation became the serious candidate [4]. Derivatives of the BOC concept are also utilized by the GNSSs, such as the alternative BOC (AltBOC) for E5 Galileo and B2 Beidou, multiplexed BOC (MBOC) for E1 Galileo and B1 Beidou, time-multiplexed BOC (TMBOC) for L1 GPS. Although these modulation schemes offer significant performance in terms of tracking accuracy, multipath mitigation and jamming reduction, there are still critical issues to be addressed, such as their large spectrum occupancy, high spectral side lobes and implementation complexity [5,6]. Subsequent innovations for signal design have focused on the continuous phase modulation (CPM), multilevel coded symbol (MCS) and m-PSK BOC modulation [7,8,9] approaches, where there is also a trade-off between implementation complexity and signal performance.

Multi-carrier signals are widely used in terrestrial communication, and even in satellite communication. The study of multi-carrier signals demonstrates they have efficient spectrum utilization, high out-of-band attenuation properties and multipath mitigation capability. Especially, orthogonal frequency division multiplexing (OFDM), as a representative, has already been the dominant modulation scheme for mobile communications. For example, the fourth generation (4G) mobile communication adopts OFDM as its core technology [10]. The European digital video broadcast-terrestrial (DVB-T) system utilizes OFDM modulation as the air interface [11]. The OFDM-based terrestrial communication signals have become a signal-of-opportunity candidate for positioning services to supplement GNSS [12,13]. Thus, multi-carrier modulation possesses potential navigation signal capability and enables integrated communication and navigation services. 

The use of multi-carrier-based signals for navigation applications is still in an incipient stage. There are several tentative modulation schemes, such as filtered multi-tone (FMT) [14,15], OFDM [16,17], or time-division orthogonal frequency division multiplexing (TD-OFDM) [18]. In this paper, we propose a novel modulation scheme called navigation signal based on compound carrier (NSCC). The NSCC concept, based on a composite design of navigation signals and communication signals, realizes the integration of navigation and communication. The novel signal possesses a large set of constituent parameters, which makes it display great design freedom. The excellent capability inherited from the multi-carrier signal approach, render it a viable future GNSS signal mode, especially as a promising navigation augmentation signal.

Moreover, the NSCC signal enables the integration of navigation and communication services, and hence strengthens the combination of GNSSs with ubiquitous terrestrial positioning systems, and above all, with communication systems at the signal level. This integrated signal will support and even spawn new applications, referred to as location-aware or location-based services (LBSs) [19,20].

A preliminary exploration has been conducted to confirm the feasibility of the proposal [21]. The NSCC, however, has a serious shortcoming with its high peak to average power ratio (PAPR) due to its multi-carrier structure. Studies show that constant envelope techniques and PAPR restraint techniques can be used to address this problem [22,23].

The paper is organized as follows: firstly, the concept and principle of the NSCC signal are briefly introduced, and the general mathematical expressions of its auto-correlation function (ACF) and power spectral density (PSD) are presented. Secondly, comprehensive evaluation criteria and analytical methods are provided in terms of spectral efficiency, tracking accuracy, anti-jamming reduction and multipath mitigation capability. The related merits are used to quantify the performance of NSCC and the legacy modulation schemes. Thirdly, the proposed NSCC signal methd together with the existing navigation signals are comprehensively evaluated and analysed. Finally, the main performance features of the novel signal are discussed and our conclusions presented.

## 2. NSCC and Its Properties

### 2.1. Mathematical Model

The multicarrier-based NSCC signal mainly realizes the function of pseudo-range measurement with carrier phase and spread spectrum code phase, as well as transmitting the low rate integration navigation and communication messages. For the *i*-th transmitter, the time-domain mathematical expression is given by:

(1) $$S^{i}\left(t\right)=\sum_{m=1}^{M}A_{m}^{i}C_{m}^{i}\left(t\right)D_{m}^{i}\left(t\right)\cos\left(2\pi \left(f_{0}+\Delta f_{m}\right)t+\varphi_{m}\right)$$

where, *M* is the number of subcarriers, the index *m* ∈ [1.M] refers to the m-th subcarrier, *A_m_* is the amplitude of the m-th subcarrier, *C_m_* is the pseudo-random noise (PRN) spreading code sequence for the m-th subcarrier, *D_m_* is the navigation data for the m-th subcarrier, if any, *f*_0_ is the initial frequency, Δ*f_m_* is the frequency interval between the m-th subcarrier and the initial frequency; *ϕ_m_* is the initial phase of the m-th subcarrier. Furthermore, the transmitters are distinguished by the PRNs sets. As for sub-carriers of the NSCC signal, the PRNs and frequency orthogonality can be utilized to suppress the inter-channel interference (ICI).

As mentioned above, the NSCC signal has a large set of constituent parameters, such as the number of subcarriers, the subcarrier frequency interval, the power distribution and the subcarrier PRNs. These structural parameters provide large degrees of freedom and flexibility for signal design. The parameter configuration is generally adjusted according to constraints and requirements. Therefore, in order to optimize the GNSS receiver performance, a judicious choice of these parameters is necessary.

Furthermore, according to the independence of its sub-carriers, the NSCC signal can split its PSD flexibly in order to avoid deliberate interferences and coexist with the legacy signals sharing the same frequency bandwidth. Thus, the split spectrum characteristic is its distinct superiority feature.

For the sake of simplicity, we set up two basic NSCC configurations for the reference baseline compared with the legacy navigation signals. The so-called baseline is based on a monomodal and a bimodal structure which are extracted from the features of the legacy navigation signals. The monomodal structure concentrates the sub-carriers near the bandwidth center, and the bimodal structure distributes the sub-carriers symmetrically near the bandwidth edges.

### 2.2. ACF and PSD

The important basic characteristics NSCC processes are presented in terms of ACF and PSD. The signal ACF should have a narrow peak to realize precise range measurement. For efficient spectral utilization, the signal PSD should possess a narrow profile to allow the co-existence of a number of other signals with minimal mutual interference.

In essence, the NSCC is the sum of the *M* individual subcarriers with BPSK modulation. The normalized PSD for NSCC can be expressed as:

(2) $$G_{NSCC}\left(f\right)=\frac{1}{M}\sum_{m=0}^{M-1}f_{c}{\left|\frac{\sin\left[\pi \left(f-f_{m}\right)T_{c}\right]}{\pi \left(f-f_{m}\right)}\right|}^{2}$$

where, *f_m_* is the m-th subcarrier frequency, *f_c_* is the chip rate of spread spectrum PRN code modulating the subcarriers, *T_c_* is the symbol period.

According to the Wiener-Khintchine theorem，a signal ACF function and its PSD function constitute a Fourier transform pair. Therefore, the NSCC ACF can be derived from the inverse Fourier transformation of its PSD:

(3) $$R_{NSCC}\left(t\right)=\int_{-B_{r}/2}^{B_{r}/2}\frac{1}{M}\sum_{m=0}^{M-1}f_{c}{\left|\frac{\sin\left[\pi \left(f-f_{m}\right)T_{c}\right]}{\pi \left(f-f_{m}\right)}\right|}^{2}e^{j2\pi ft}df$$

where, *B_r_* stands for the pre-filtering bandwidth of GNSS receiver.

**Figure 1:**
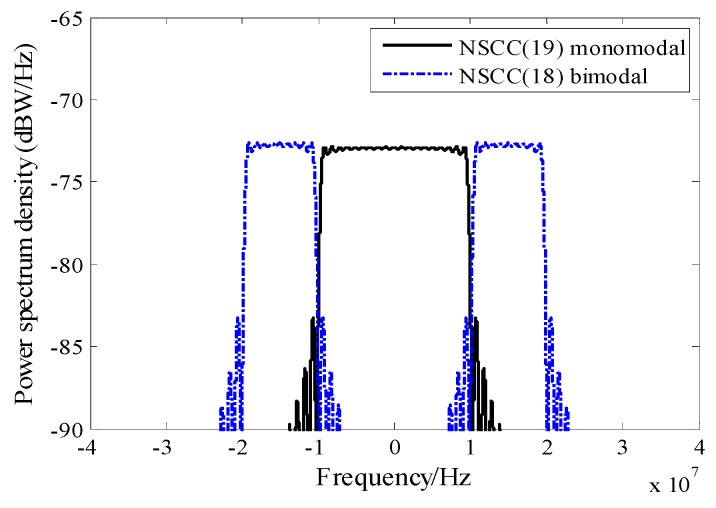
Comparison of PDSs for NSCC baseline structures.

The PSDs of the monomedal and the bimodal NSCC signal are illustrated in Figure 1. The baseline structures adopt a constant power distribution within the active subcarriers. Their subcarrier configuration selects BPSK modulation, 1.023 Mcps code rate and 1.023 MHz frequency spacing. The number of sub-carriers is 19 for monomodal and 18 for bimodal mode with a similar spectrum content over the 20 MHz bandwidth.

The two baselines occupy the same bandwidth and can coordinate the spectrum with preexisting signals over the shared bandwidth. Note that their OOB emissions are nearly equal due to the similar sub-carrier configurations. 

**Figure 2:**
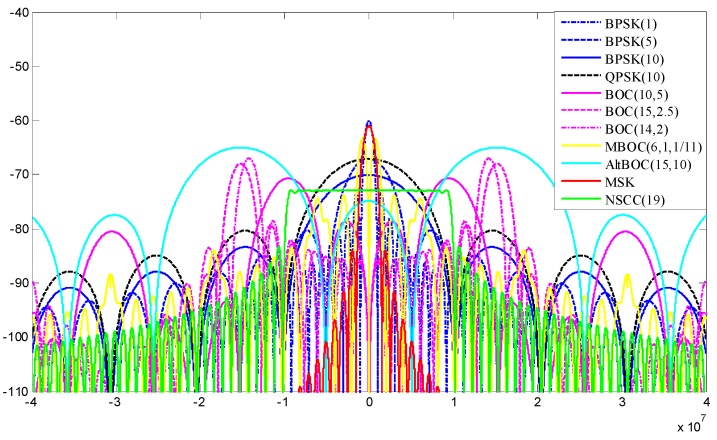
Comparison of PSDs for NSCC and legacy navigation signal modulations.

The PSDs of NSCC and legacy navigation signals are shown in Figure 2. The profiles of signal PSDs reflect the spectral efficiency and adjacent channel interference (ACI) suppression properties. To illustrate the details of the PSD profiles, the relative power attenuations between the first side lobe and the main lobe are given in Table 1. The modulations include BPSK(1), BPSK(10), BOC(10,5), QPSK(10) used in GPS, BPSK(5), MBOC(6,1,1/11), BOC(10,5), BOC(15,2.5), AltBOC(15,10) used in Galileo, BOC(15,2.5), BOC(14,2), MBOC(6,1,1/11), QPSK(10) used in Beidou system. Only monomodal NSCC is adopted because of the similarity of spectral characteristics with the bimodal one except for the split feature.

**Table 1:** The relative power attenuations between the first side lobe and the main lobe.

Modulation Scheme	Main Lobe (dBW/Hz)	First Side Lobe (dBW/Hz)	Power Attenuation (dBW/Hz)
BPSK(1)	−60.10	−73.36	13.26
BPSK(5)	−67.09	−80.35	13.26
BPSK(10)	−70.10	−83.36	13.26
QPSK(10)	−67.09	−80.35	13.26
BOC(10,5)	−70.07	−78.64	7.94
BOC(15,2.5)	−67.96	−79.32	11.36
BOC(14,2)	−67.00	−78.61	11.61
BOC(6,1,1/11)	−63.31	−73.87	10.56
AltBOC(15,10)	−64.99	−74.87	9.88
MSK	−61.01	−84.01	23.00
NSCC	−70.10	−82.05	12.04

Obviously, the energy of the NSCC signal is mostly concentrated within the main lobe and the magnitude of the side lobes decreases sharply. The NSCC modulation makes the PSD more compact than BOC modulation and its derivatives. This property implies better spectrum efficiency and less vulnerability to adjacent channels. We note that the MSK signal presents the best spectrum efficiency and the side lobes fall off sharpest because of its constant envelope modulation, but the relatively smaller bandwidth is a restriction for the ultimate performance.

**Figure 3:**
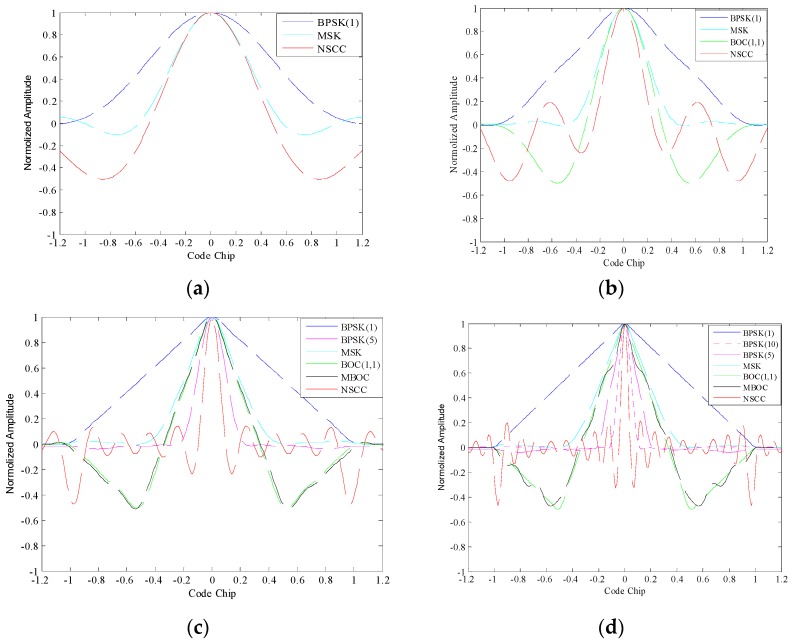
ACFs for NSCC modulation and legacy navigation modulations with: (**a**) 2 MHz, (**b**) 4 MHz, (**c**) 10 MHz, (**d**) 20 MHz pre-filtering bandwidths.

The ACFs of NSCC and the legacy signals are shown in Figure 3. A signal ACF profile is closely related to its bandwidth since the high-frequency component determines the details. The simulation bandwidths are set as 2, 4, 10 and 20 MHz, respectively. The monomodal NSCC is selected for comparison in the simulation.

For the 2 MHz bandwidth, the MSK modulation possesses a narrower ACF peak than BPSK(1) modulation because its main lobe concentrates more power within 2 MHz. Then, the BOC(1,1) modulation outperforms the MSK modulation at 4 MHz bandwidth due to the more high-frequency components it possesses. With the increment to 10 MHz, the ACF profiles of BOC(1,1) become steeper, but the MBOC modulation doesn’t appear to have any advantage compared with BOC(1,1) resulting in the loss of high-frequency components. Note that the NSCC modulation presents superior tracking performance with the narrowest ACF profiles in comparison with legacy navigation signals, and the NSCC ACF become increasingly steep as the pre-filtering bandwidth increases, closely followed by the BPSK(5) at 10 MHz bandwidth and BPSK(10) at 20 MHz bandwidth.

**Figure 4:**
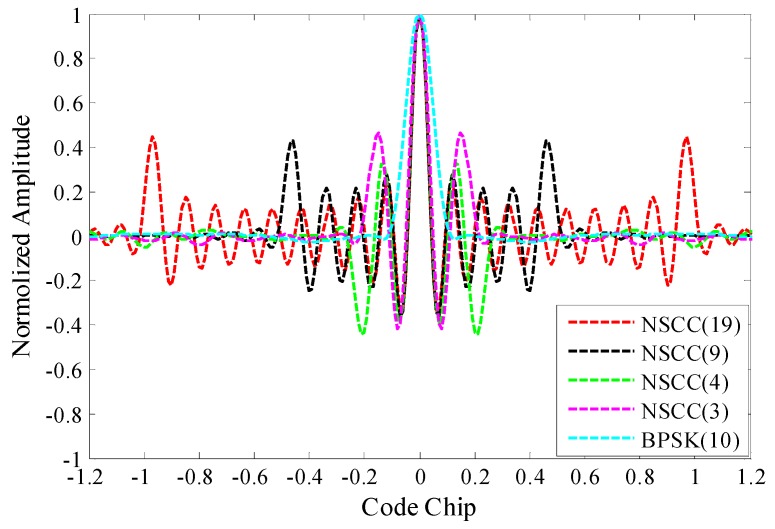
ACFs for monomodal NSCCs with different sub-carriers in 20 MHz bandwidth.

**Figure 5:**
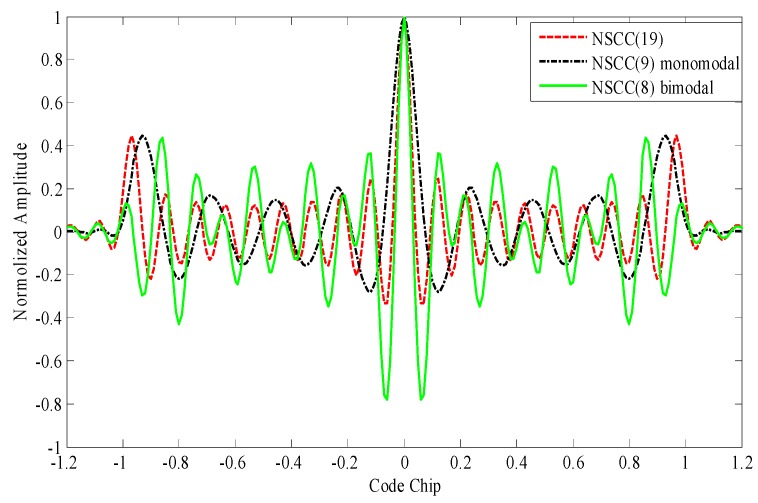
Comparison of ACFs for NSCC baseline structures within 20 MHz.

We further investigate the ACFs for NSCCs with different parameter configurations including the number of sub-carriers, the subcarrier chip rate, the subcarrier spacing and the power distribution. The results are shown in Figure 4 and Figure 5.

Figure 4 illustrates the ACFs for monomodal NSCCs within 20 MHz pre-filtering bandwidth. The NSCCs are allocated constant power distribution. The code rate is 1.023 Mcps for NSCC(19), 2.046 Mcps for NSCC(9), 4.092 Mcps for NSCC(4), 5.115 Mcps for NSCC(3), 10.23 Mcps for BPSK(10). The number of sub-carriers are set up in conjunction with the chip rate to adapt the finite bandwidth. It can be seen that as the number of sub-carriers increases and the chip rate reduces, the sharpness of the main peaks of the NSCCs is enhanced slightly and the locations of side-lobes move gradually far away from the main peaks.

Figure 5 shows the ACFs for NSCC(19), NSCC(9) monomodal and NSCC(8) bimodal. These signals all adopt 1.023 MHz chip rate, 1.023 MHz sub-carrier spacing and constant power distribution. The main-lobes of the two baselines have no overlap with each other in the frequency domain. Note that the largest proportions of high-frequency component enhance the second order moment of the spectrum, rendering the NSCC signal to process sharpest peaks, while it also brings to the highest secondary lobes and these locations are closest to main peak.

Besides, the NSCC has an obvious drawback due to its multi-peak ACF. Its characteristic ACF relates to the frequency information contained within the NSCC baseband signal, and the secondary lobes far to the zero delay are associated with the superposition of NSCC subcarriers’ ACFs. The abovementioned feature implies possible false acquisition or biased tracking, resulting in ambiguous measurements if no special care is taken. Since the NSCC is essentially the superposition of subcarriers with BPSK modulation, the conventional BPSK techniques can be utilized to avoid the ambiguity, and above all, be combined with the NSCC subcarrier information to promote their performance [24,25]. Another solution is the bump and jump (BJ) technique, proposed by Fine and Wilson in [26], which uses extra correlators referred to as very early (VE) and very late (VL) correlators to monitor the amplitudes of the suspicious side peaks.

## 3. Evaluation Criteria

This section presents the analytical methods and evaluation criteria applied to quantify and assess the signal performance and provide essential references for signal design.

### 3.1. Spectral Separation Coefficient

Mutual interference and compatibility with GNSS signals sharing the same frequency band, are the fundamental factors for the frequency coordination [27]. The Spectral Separation Coefficients (SSCs) represent the conventional approach to indicate and evaluate the degradation caused by the interference signals at the correlator output [28]. Thus, the correlator output can be expressed as:

(4) $$P_{o}={\left\|\int_{-\infty }^{\infty }\left\{\sqrt{P_{i}}S_{i}\left(f\right)+\sqrt{P_{d}}S_{d}\left(f\right)\right\}H\left(f\right)d\text{​}f\right\|}^{2}$$

where, the *P_d_* and *P_i_* refer to the power level of the desired signal and the interfering signal respectively, *S_d_*(*f*) and *S_i_*(*f*) refer to the spectrum of the desired signal and the interfering signal, *H*(*f*) is an ideal matched filter, thus equals to the conjugate of the desired signal spectrum.

Assuming that the cross-spectrum term is zero due to the non-correlation between the desired signal and the interfering signal. Then, the Equation (4) can be simplified as:

(5) $$P_{o}=P_{i}\int_{-\infty }^{\infty }G_{i}\left(f\right)G_{d}\left(f\right)df+P_{d}\int_{-\infty }^{\infty }G_{d}^{2}\left(f\right)df$$

where, the terms *Gs*(*f*) and *Gi*(*f*) stand for the normalized PSDs of the desired and interfering signals, respectively. The SSCs can thus be expressed as the following form:

(6) $$\kappa_{ss}=\int_{-\infty }^{\infty }G_{s}^{2}\left(f\right)df$$

(7) $$\kappa_{si}=\int_{-\infty }^{\infty }G_{i}\left(f\right)G_{s}\left(f\right)df\;$$

Clearly, *κ_ss_* and *κ_si_* quantify a signal’s self-imposed SSC and mutual interference SSC, respectively.

### 3.2. Tracking Performance

The Cramer-Rao Lower Bound (CRLB) is the generic theorem used to assess the theoretical accuracy of time-delay estimation. It defines the ultimate accuracy and sets the physical limit of the signal in white noise with given bandwidth for any estimate of a non-random parameter [29,30]. The CRLB is given as:

(8) $$CRLB=\frac{B_{L}}{C/N_{0}{\left(2\pi \right)}^{2}\int_{-B_{r}/2}^{B_{r}/2}f^{2}G\left(f\right)d\text{​}f}$$

where, *B_L_* refers to the single-sided equivalent bandwidth of the code tracking loop, *C*/*N*_0_ is the ratio of carrier power to noise density, *G*(*f*) is the normalized PSD of the signal over the front-end bandwidth *B_r_*. For convenience, the Gabor bandwidth, defined as the root of the second moment of the signal PSD, is employed to be an alternative interpretation of CRLB. Its expression is:

(9) $$f_{Gabor}=\sqrt{\int_{-B_{r}/2}^{B_{r}/2}f^{2}G\left(f\right)d\text{​}f}$$

Therefore, the greater Gabor bandwidth, the better the potential code-tracking accuracy will be.

### 3.3. Anti-Jamming Capability

The effective methodology to predict and access the anti-jamming capability is based on the effective carrier to noise density ratio ((*C/N_0_*)*_eff_*) [31]. The quantity (*C/N_0_*)*_eff_*, indicates the level of interference on characteristics at the input of the receiver. According to the representative of the GNSS receiver functions and the interference characteristics, the following merits factors are adopt to describe the anti-jamming performance [32,33]:

The Demodulation & anti-jamming of narrowband (Dem&AJNB) merit factor is given by:

(10) $$Q_{DemAJNB}=10\mathrm{lg}\left(\frac{1}{R_{d}\cdot \max\left[G_{s}\left(f\right)\right]}\right)\left(\text{dB}\right)$$

where, *R_d_* is the message rate, max[·] represents the maximum operation.

The Code Tracking & anti-jamming of narrowband (CT&AJNB) merit factor is given by:

(11) $$Q_{CTAJNB}=10\mathrm{lg}\left(\frac{\int_{-\beta_{r}/2}^{\beta_{r}/2}f^{2}G_{s}\left(f\right)df}{\max\left[f^{2}G_{s}\left(f\right)\right]}\right)\left(\text{dB}\right)$$

The Demodulation & anti-jamming of matched spectrum (Dem&AJMS) merit factor is given by:

(12) $$Q_{DemAJMS}=10\mathrm{lg}\left(\frac{1}{R_{d}\cdot \int_{-\beta_{r}/2}^{\beta_{r}/2}G_{s}^{2}\left(f\right)\text{​}df}\right)\left(\text{dB}\right)$$

The Code Tracking & anti-jamming of matched spectrum (CT&AJMS) merit factor is given by:

(13) $$Q_{CTAJMS}=10\mathrm{lg}\left(\frac{\int_{-\beta_{r}/2}^{\beta_{r}/2}f^{2}G_{s}\left(f\right)\text{​}df}{\int_{-\beta_{r}/2}^{\beta_{r}/2}f^{2}G_{s}^{2}\left(f\right)\text{​}df}\right)\left(\text{dB}\right)$$

The greater the quality factors, the better the anti-jamming capability will be. It should be noted that the performance of the anti-jamming of the narrow band interference largely depends on the maximum of the normalized PSD of a signal, that is, the intentional narrow band interference aims at the maximum power of the signal spectrum to achieve the interference of utility-maximization. The performance of the anti-jamming of the matched spectrum interference depends greatly on the degree of overlap of the signals.

### 3.4. Multipath Error Envelope

The multipath error envelope (MPEE) commonly relies on the assumption of a multiple ray signal model to quantify the worst possible multipath-induced bias. The envelope graphs, as a significant criteria, directly illustrate the differences of the multipath performance for modulation schemes [34].

The two-ray signal model allowing for the direct line of sight (DLOS) signal and one single multipath signal, visualizes the common multipath environment. In this dedicated model, the output S-curve of the Early-Minus-Late (EML) discriminator can be expressed as [35]:

(14) $$\begin{array}{ll} D_{EML}\left(\varepsilon \right) & =\left[R\left(\varepsilon +d/2\right)-R\left(\varepsilon -d/2\right)\right]\\ & +a\cos\left(\Delta \varphi \right)\left[R\left(\varepsilon -\Delta \tau_{1}+d/2\right)-R\left(\varepsilon -\Delta \tau_{1}-d/2\right)\right] \end{array}$$

where *ε* is the code delay estimation error, *d* is the correlator spacing between the early and late reference signal, *R* is the correlator function of the received signal and reference signal, *a* is the multipath signal to DLOS amplitude ratio (MDR), Δ*ϕ* is the phase difference between the multipath and the DLOS, Δ*τ*_1_ is the multipath signal time delay refer to DLOS. Note that the multipath error envelops are computed using the two extreme cases, where Δ*ϕ =* 180° and 0°.

Expanding the *D_EML_*(*ε*) with the first-order Taylor’s series approximation about 0, Equation (14) becomes:

(15) $$D_{EML}\left(\varepsilon \right)\approx D_{EML}\left(0\right)+D_{EML}^{\prime }\left(0\right)\times \varepsilon +o\left(\varepsilon^{2}\right)$$

where *o*(*ε*^2^) represents higher-order errors. Neglecting the influence of higher-order terms, Equation (15) can be expressed as:

(16) $$\varepsilon \approx -D_{EML}\left(0\right)/D_{EML}^{\prime }\left(0\right)$$

According to the relationship between the ACF and PSD of a signal, the MPEE can be obtained as:

(17) $$\varepsilon \approx \frac{\pm a\int_{-B_{r}/2}^{B_{r}/2}G\left(f\right)\sin\left(2\pi f\tau \right)\sin\left(\pi f\;d\right)df}{2\pi \int_{-B_{r}/2}^{B_{r}/2}fG\left(f\right)\sin\left(\pi f\;d\right)\left[1\pm a\cos\left(2\pi f\tau \right)\right]df}$$

where, the operation symbols “+” and “−” correspond to the aforementioned two extreme cases, respectively. From Equation (17), we can obtain that the MPEE lobes strictly depend on the characteristics of the signal ACF and PSD. The sharper peak of a signal ACF means more high-frequency components a signal PSD processes, then, the corresponding MPEE will be smaller and decrease faster towards zero.

The related average multipath error envelop (AMPEE) expressions are given by:

(18) $$\varepsilon_{av}=\frac{1}{\tau }\int_{0}^{\tau }\left[\frac{abs\left(\varepsilon {|}_{\Delta \varphi =0}\right)+abs\left(\varepsilon {|}_{\Delta \varphi =180}\right)}{2}\right]\;d\tau$$

The AMPEE reflects the susceptibility to multipath interference at any delay.

## 4. Evaluation and Analysis of NSCC with Representative Parameters

### 4.1. Spectral Separation Coefficient

The SSCs for typical modulation schemes are listed in Table 2. The NSCC modulation adopts flat power distribution for sub-carriers, and the number of mutually orthogonal sub-carriers is 19. The pre-filtering bandwidth used in the simulation is 20 MHz.

The comparison data in the Table 2 demonstrate that the NSCCs possesses better SSCs than P, MBOC(6,1,1/11), BOC(1, 1), MSK, C/A, except for NSCC monomodal. This is because the NSCC signals consist of spectrally overlapping and mutually orthogonal sub-carriers resulting in very efficient spectrum utilization. The NSCC monomodal signal concentrates its main power within a 10 MHz bandwidth centered on the carrier frequency, yielding the largest overlap with the legacy navigation signals, while the NSCC bimodal dodges these legacy signals and imposes the least interference on the desired signals. Furthermore, with the increasing chip rate, the compatibility between NSCC modulation and C/A, MSK, BOC(1,1) MBOC(6,1,1/11), is becoming worse, but for larger bandwidth signals, such as QPSK(10), P, the compatibility is improving. It is not surprising that the number of subcarriers associated with the chip rate under the limitation of finite bandwidth, influences the power lever for NSCC with equipower distribution.

**Table 2:** SSC values for BPSK, QPSK, BOC, MBOC, MSK and NSCCs for 20 MHz pre-filtering bandwidth.

SSCs(dB)	C/A	MSK	BOC(1,1)	MBOC(6,1,1/11)	QPSK(10)	P	NSCCself
C/A	−61.9						
MSK	−62.3	−62.4					
BOC(1,1)	−67.9	−66.3	−64.9				
MBOC(6,1,1/11)	−68.3	−66.7	−65.3	−65.7			
QPSK(10)	−67.2	−67.1	−67.5	−67.9	−65.9		
P	−70.2	−70.1	−70.6	−70.9	−68.9	−71.9	
NSCC(19)	−73.0	−72.9	−73.1	−73.1	−70.4	−73.4	−73.1
NSCC(9)	−72.9	−72.7	−73.1	−73.5	−71.2	−74.2	−73.0
NSCC(4)	−72.6	−72.5	−73.1	−73.5	−73.5	−76.5	−72.9
NSCC(3)	−72.5	−71.9	−73.0	−73.4	−74.5	−77.5	−72.7
NSCC Mono	−69.8	−69.7	−70.1	−70.5	−68.2	−71.2	−70.0
NSCC Bimo	−87.0	−89.9	−84.3	−79.6	−75.7	−78.7	−69.8

### 4.2. Tracking Performance

**Figure 6:**
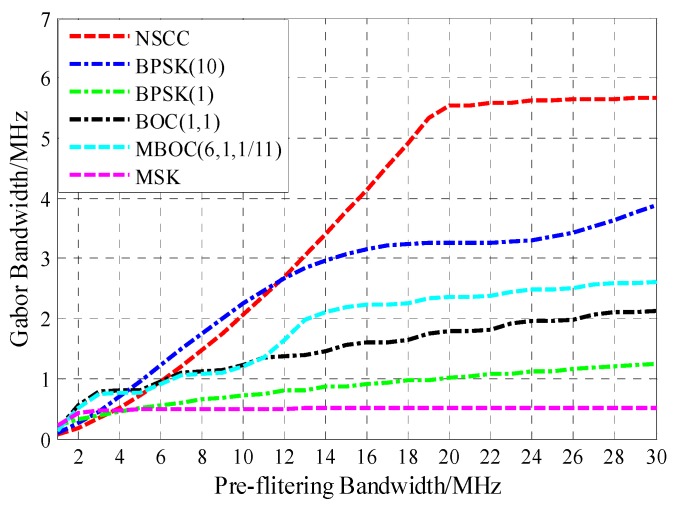
Gabor bandwidth of the legacy navigation signals and NSCC modulation.

With the front-end bandwidth ranging from 0 to 30 MHz, the Gabor bandwidth is computed and the corresponding result is illustrated graphically in Figure 6.

Figure 6 shows that when the front-end bandwidth is <6 MHz, the Gabor bandwidth of NSCC(19) is lower than that of the BPSK(10), BOC(1,1) and MBOC(6,1,1/11) modulation. As the front-end bandwidth increases, the Gabor bandwidth becomes large until 20 MHz, which is the main lobe bandwidth for NSCC(19). The Gabor bandwidth of NSCC outperforms BPSK(10) until the pre-filtering bandwidth approximately equals 12 MHz. For the very large front-end bandwidth, NSCC presents the best code-tracking performance. Therefore, the NSCC shows the best code-tracking performance among the analyzed signals for large pre-filtering bandwidths.

**Figure 7:**
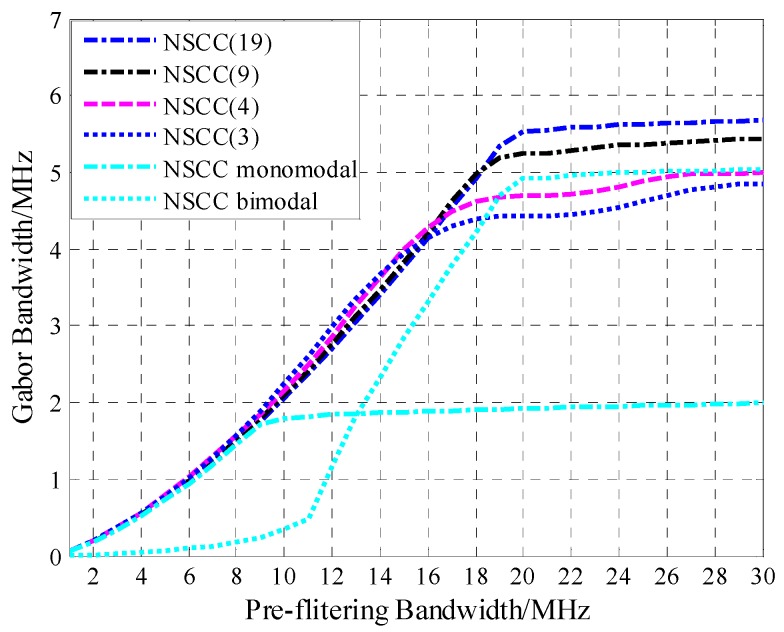
Gabor bandwidth for NSCCs.

Figure 7 illustrates the Gabor bandwidth for NSCC modulation with different parameter configurations. Obviously, the NSCCs Gabor bandwidths all appear to be approaching their peak values above 20 MHz, which is the main lobe bandwidth of the NSCCs for our simulation. Note that the less the chip rate, the greater the Gabor bandwidth peak value is. As for the reference baseline, the NSCC bimodal presents excellent code-tracking performance compared favorably with NSCCs occupied 20 MHz bandwidth, while the NSCC monomodal just gets approximately half of the bimodal Gabor bandwidth.

### 4.3. Anti-Jamming Capability

**Figure 8:**
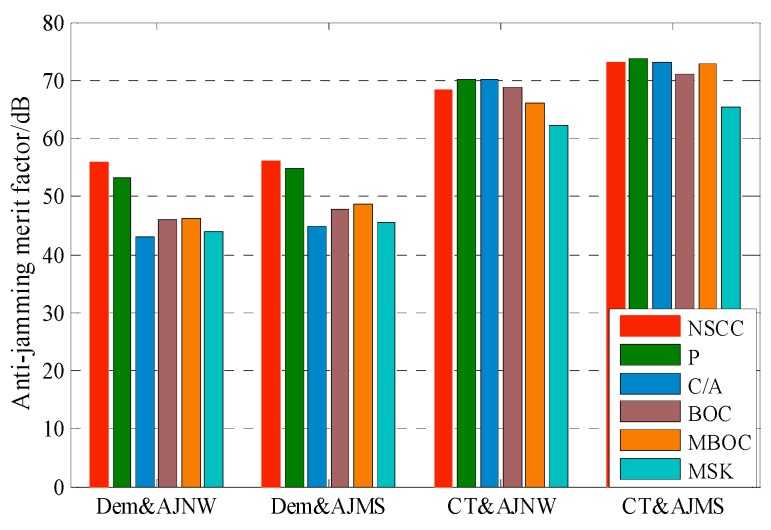
The comparison of anti-jamming merit factor for 20 MHz pre-filtering bandwidth.

Figure 8 shows the numerical result for anti-jamming merit factors of NSCC and legacy signals. Clearly, the NSCC presents superior Dem&AJNB and Dem&AJSM performance among the signals analyzed due to its excellent spectral efficiency and flat power distribution. Note that, the merit factor of NSCC signal is at least 2 dB greater than P code. As for anti-jamming of code tracking performance, however, the NSCC appears inferior to P code resulting from the more high-frequency components of the NSCC signal but slighter power distribution fluctuation.

**Figure 9:**
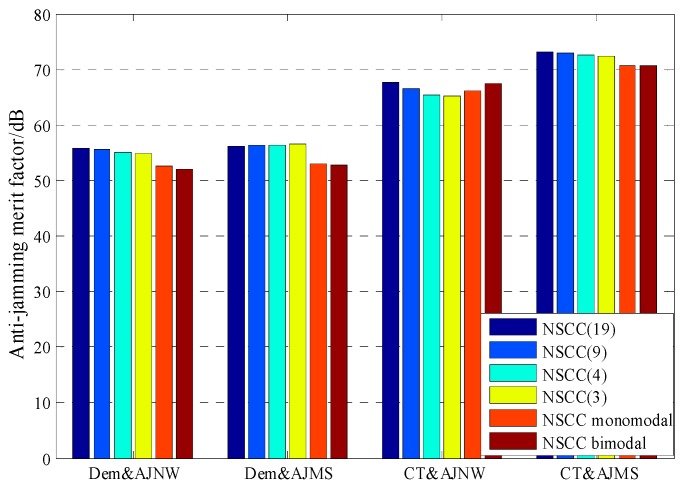
The comparison of anti-jamming merit factor for NSCCs within the 20 MHz bandwidth.

Figure 9 shows the anti-jamming merit factors of NSCC modulation within the 20 MHz pre-filtering bandwidth. Overall, the NSCCs with the different chip rates all display great anti-jamming capability for Dem&AJNW, Dem&AJMS and CT&AJMS, although there is a slight numerical difference. This means the three merit factors are not sensitive to the chip rate within the same pre-filtering bandwidth. The Dem&AJNW, Dem&AJMS and CT&AJMS performances of the two baseline signals are approximately 3 dB less than that of NSCCs due to the halving power and number of sub-carriers. The NSCC bimodal presents superior CT&AJNW performance resulting from the large proportion of high-frequency component.

### 4.4. Multipath Error Envelop

**Figure 10:**
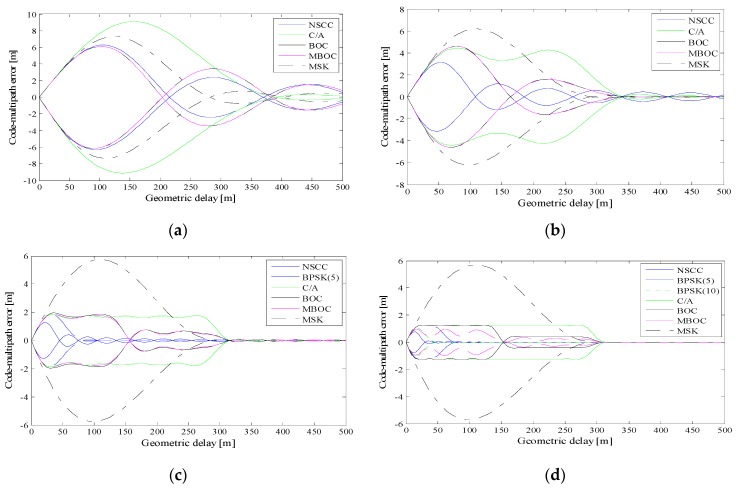
Multipath error envelops of pre-filtering bandwidth of 2 MHz (**a**), 4 MHz (**b**), 10 MHz (**c**), 20 MHz (**d**), a = 0.1, d = 0.1 chip.

Simulations were conducted to compare the multipath (MP) mitigation performance between the NSCC and other legacy satellite navigation signals. The simulation conditions are set as correlator spacing *d* = 0.1 chip, MDR *a* = 0.1. Figure 10a–d show the multipath error envelopes with 2, 4, 10, 20 MHz pre-filtering bandwidths, respectively. 

According to the Nyquist Sampling Theorem, we computed the envelopes of a signal only when the pre-filtering bandwidth is larger than the main lobes bandwidth. For the 2 MHz pre-filtering bandwidth, the MSK presents better multipath mitigation capability than C/A, but worse than the other modulation schemes investigated. As the pre-filtering bandwidth increases, the MPEEs of the legacy signals and the NSCC decrease. The greater bandwidth allows more high-frequency component in the EML discriminator, and the detailed component is demonstrated to improve the multipath performance. At the 20 MHz bandwidth, the NSCC possesses the smallest multipath envelope which is less than 1 m, and its MPEE decreases fastest and is even invisible when the multipath delay is larger than 25 m. Note that the MPEEs of the NSCC have more ripples compared with P code due to the more number of side-lobes of the NSCC ACF.

**Figure 11:**
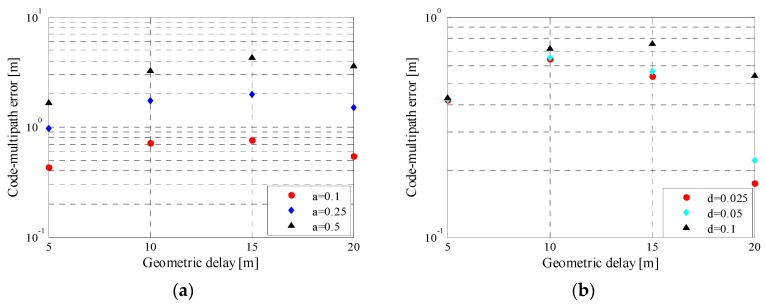
Multipath error envelop for NSCC(19) with different MDRs (**a**) and different correlator spacings (**b**).

Figure 11 shows the details about the multi-path error (MPE) for NSCC(19) with different MDRs and correlator spacings. The multipath errors are larger for the greater MDRs. This is understandable as greater multipath power will no doubt cause additional multipath power contribution. The multipath error decreases, as the correlator spacing becomes narrow. The impact of the correlator spacing is more distinct for larger geometric delay.

**Figure 12:**
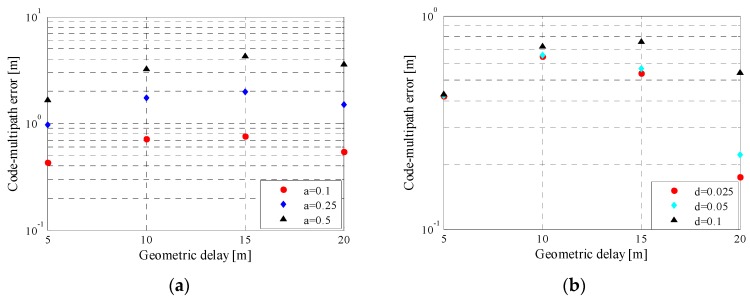
Multipath error(**a**) and average multipath error(**b**) for NSCCs with different code rates and its reference baseline.

Figure 12 shows the details for multipath-induced error and average multipath error for NSCC modulation. The MPE for NSCCs increases slightly as the chip rate grows, while AMPEs for the NSCCs appear similar within the short geometric delays such as <10 m, and are diverging visibly above 10 m path delay. According to the overall tendency of AMPE curves, the NSCCs with lower chip rate present better multipath performance which coincides with the MPE. 

The MPE and AMPE for the two baselines also diverge evidently. This indicates that a large proportion of the high-frequency components is a prominent factor to improving multipath mitigation capability within a limited bandwidth.

## 5. Conclusions 

In this paper, the NSCC modulation scheme was investigated. Its mathematical expression is presented to illustrate the signal structure and parameters. According to the NSCC modulation characteristics, we investigated the suitability for satellite navigation or navigation augmentation purposes through comparisons with the current GPS, Galileo and Beidou signals. The signal ACF and PSD were analyzed and show that the NSCC ACF profile possesses the narrowest peak to allow precise pseudo-range measurement, and its PSD is more compact over the frequency range and lower side lobes, exhibiting an efficient spectrum utilization. The NSCC modulation possesses a large set of constituent parameters, confering it a great degree of design freedom and full spectral flexibility. For its structure characteristics and scenario constraints, a reference baseline consisting of a monomodal and bimodal signal is identified.

Furthermore, the NSCC signal, together with legacy navigation signals, are comprehensively evaluated, employing the performance of interference, tracking accuracy and multipath mitigation respectively in terms of SSCs and anti-jamming quality factor, Gabor bandwidth and multipath error envelope. Both theoretical analysis and simulation show that the NSCC signal possesses less SSCs because of its efficient spectral utilization, to allow the coexistence of a large number of signals with minimal mutual interference. In addition, the tracking performance and multipath mitigation capability with the NSCC modulation scheme outperforms the other signal modulations. The high-frequency component can greatly improve both performances with same pre-filtering bandwidth, but it is not sensitive to the chip rate and number of subcarriers. The NSCC signal exhibits excellent anti-jamming reduction, while it is inferior to P code in the aspect of CT anti-jamming ability due to its flat power distribution. In conclusion, the NSCC modulation scheme provides viability for potential GNSS signal modulation schemes, especially for ground-based navigation augmentation systems.
